# Identifying COVID-19 Severity-Related SARS-CoV-2 Mutation Using a Machine Learning Method

**DOI:** 10.3390/life12060806

**Published:** 2022-05-28

**Authors:** Feiming Huang, Lei Chen, Wei Guo, Xianchao Zhou, Kaiyan Feng, Tao Huang, Yudong Cai

**Affiliations:** 1 School of Life Sciences, Shanghai University, Shanghai 200444, China; hfm123@shu.edu.cn; 2 College of Information Engineering, Shanghai Maritime University, Shanghai 201306, China; lchen@shmtu.edu.cn; 3 Key Laboratory of Stem Cell Biology, Shanghai Jiao Tong University School of Medicine (SJTUSM) and Shanghai Institutes for Biological Sciences (SIBS), Chinese Academy of Sciences (CAS), Shanghai 200025, China; gw_1992@sjtu.edu.cn; 4 Center for Single-Cell Omics, School of Public Health, Shanghai Jiao Tong University School of Medicine (SJTUSM), Shanghai 200025, China; zhouxch1@shanghaitech.edu.cn; 5 Department of Computer Science, Guangdong AIB Polytechnic College, Guangzhou 510060, China; kyfeng@gdaib.edu.cn; 6 Bio-Med Big Data Center, CAS Key Laboratory of Computational Biology, Shanghai Institute of Nutrition and Health, University of Chinese Academy of Sciences, Chinese Academy of Sciences, Shanghai 200031, China; 7 CAS Key Laboratory of Tissue Microenvironment and Tumor, Shanghai Institute of Nutrition and Health, University of Chinese Academy of Sciences, Chinese Academy of Sciences, Shanghai 200031, China

**Keywords:** SARS-CoV-2, mutation, machine learning, feature selection, decision rules

## Abstract

SARS-CoV-2 shows great evolutionary capacity through a high frequency of genomic variation during transmission. Evolved SARS-CoV-2 often demonstrates resistance to previous vaccines and can cause poor clinical status in patients. Mutations in the SARS-CoV-2 genome involve mutations in structural and nonstructural proteins, and some of these proteins such as spike proteins have been shown to be directly associated with the clinical status of patients with severe COVID-19 pneumonia. In this study, we collected genome-wide mutation information of virulent strains and the severity of COVID-19 pneumonia in patients varying depending on their clinical status. Important protein mutations and untranslated region mutations were extracted using machine learning methods. First, through Boruta and four ranking algorithms (least absolute shrinkage and selection operator, light gradient boosting machine, max-relevance and min-redundancy, and Monte Carlo feature selection), mutations that were highly correlated with the clinical status of the patients were screened out and sorted in four feature lists. Some mutations such as D614G and V1176F were shown to be associated with viral infectivity. Moreover, previously unreported mutations such as A320V of nsp14 and I164ILV of nsp14 were also identified, which suggests their potential roles. We then applied the incremental feature selection method to each feature list to construct efficient classifiers, which can be directly used to distinguish the clinical status of COVID-19 patients. Meanwhile, four sets of quantitative rules were set up, which can help us to more intuitively understand the role of each mutation in differentiating the clinical status of COVID-19 patients. Identified key mutations linked to virologic properties will help better understand the mechanisms of infection and will aid in the development of antiviral treatments.

## 1. Introduction

The outbreak of novel severe acute respiratory syndrome coronavirus 2 (SARS-CoV-2) initiated the pandemic of the global infectious disease called COVID-19 [1]. The rapid spread of SARS-CoV-2 has affected more than 300 million people from over 200 countries as of January 2022, and it has severely impacted public health and social economy. Scientists have achieved some successes in vaccine development, and vaccination programs are helping to prevent the infection and spread of SARS-CoV-2. However, emerging variants of coronavirus with increased pathogenicity and infectivity are still a challenge to public health [2,3]. SARS-CoV-2, as a type of single-stranded positive RNA virus, displays a stronger ability of evolution through highly frequent genomic mutations during transmission [4]. The rapid evolution of SARS-CoV-2 has brought great challenges to epidemic prevention. Viral genomics has become a major focus of current research which aims to explore the association between genomic mutations and virological properties, such as virulence, pathogenicity, and immunogenicity. Identifying the key mutations related to virological properties will largely contribute to understanding the mechanism of infection, which is important for antiviral treatment development.

SARS-CoV-2 belongs to the *Betacoronavirus* genus of the *Coronaviridae* family, which is considered to originate from bats and can widely spread in bats, civets, and humans, and usually causes respiratory illness in humans [5]. The genome of SARS-Cov-2 consists of several nonstructural proteins (nsp), which play crucial roles in viral replication, and four main structural proteins including envelope, membrane, nucleocapsid, and spike, which are involved in the process by which SARS-CoV-2 enters human cells via interacting with the host receptor ACE2 [6,7]. During the current pandemic, five variants of concern have emerged including B.1.1.7 (Alpha), B.1.351 (Beta), P.1 (Gamma), B.1.617.2 (Delta), and B.1.1.529 (Omicron). 

The B.1.1.7 variant was first discovered in the UK in September 2020, and then it spread rapidly worldwide. The S protein of the B.1.1.7 strain has eight mutations (i.e., Δ69–70 deletion, Δ144 deletion, N501Y, A570D, P681H, T716I, S982A, and D1118H), which may have changed the transmission and infection ability of SARS-CoV-2 [8]. Nine mutations (i.e., L18F, D80A, D215G, R246I, K417N, E484K, N501Y, D614G, and A701V) were found in the S protein of the B.1.351 variant [9]. The E484K site located in the receptor-binding domain (RBD) of the S protein directly contacts human ACE2 residue, which reduces the neutralization susceptibility to convalescent serum. The P.1 variant has 10 mutations in S protein (i.e., L18F, T20N, P26S, D138Y, R190S, H655Y, T1027I V1176, K417T, E484K, and N501Y), among which 3 mutations (K417T, E484K, and N501Y) show striking similarity to the RBD of the B.1.351 variant [10]. The B.1.617.2 variant was first discovered in India in October 2020 and caused a second fatal wave of COVID-19 infections in India in April 2021, which quickly spread and attracted global attention. Its transmissibility has increased by 97% compared with that of previous circulating strains [11]. Three key mutations in the S protein of the B.1.617.2 strain (i.e., L452R, T478K, and P681R) reduce the probability of reinfection and the effectiveness of the vaccine. The genome of the Omicron variant strain has about 50 mutations, including more than 30 mutations in the S protein, which overlaps with the B.1.617.2 and B.1.1.7 variants [12]. Previous studies have shown that K417N, E484K, and N501Y mutations in the S protein exhibit the enhanced immune evasion ability of Omicron [13]. The S protein is thought to be the most critical affecting factor of the virulence and pathogenicity of SRAS-CoV-2.

The implications of SARS-CoV-2 genomic mutations on viral pathogenicity have been summarized in some reviews [14]. However, the impact of viral mutations on COVID-19 severity demands further exploration. Previous articles have also mainly focused on mutations in the S protein and drawn conclusions through clinical research of limited cohort sizes. Identifying the potential mutational risk that indicates disease severity will be important for medical care and targeted treatment. To this end, we integrate the available data describing SARS-CoV-2 variants and patients’ outcomes in this study and perform a novel computational analysis to investigate the influences of mutations in the SARS-CoV-2 genome on COVID-19 severity. Mutations in the whole genome, including structural proteins, nonstructural proteins, and untranslated regions (UTRs), were included. Several advanced computational methods were applied in the feature selection process. The associations of genomic mutations of SARS-CoV-2 with viral pathogenicity and patients’ clinical outcome were revealed. Many mutations in the S protein of remarkable relevance to clinical outcome were obtained, such as V1176F and D614G. Several genomic mutations in the nucleocapsid gene or nonstructural proteins were also closely related to the severity of COVID-19 upon infection, which may indicate altered viral pathogenicity. This study provides a novel computational approach to identify new potential mutations related to viral pathogenicity. The key mutations identified in this study provide the theoretical basis of pathogenesis, and these results will impact the development and application of antiviral vaccines.

## 2. Materials and Methods

### 2.1. Data and Preprocessing

We downloaded 1513 viral genomes from the GISAID database (https://www.gisaid.org/ accessed on 6 December 2021), and the accession numbers were provided by Nagy et al. [15]. Each patient of viral origin was accompanied by clinical follow-up data. Nagy et al. divided 716 of these patients of viral genomic origin into the “mild” group and the 797 others into the “severe” group [15]. To investigate the differences between two groups, a binary classification problem was set up where patients in the “mild” group were termed as positive samples and those in the “severe” group were considered as negative samples. Most of the patients in the severe group were in the ICU, and some had even died. The majority of patients in the mild group were stable, and some were asymptomatic so could be treated at home. Mutation features contained two main types: one was protein mutations, and the other was nonprotein mutations. The protein mutations were extracted from the analysis program of the Coronavirus Antiviral Research Database (https://covdb.stanford.edu/ accessed on 6 December 2021) [16]. This analysis program was fed viral sequences in FASTA format as the input, using “Wuhan-Hu-1(NC_045512.2)” as the reference sequence. The nonprotein mutations were extracted using the MUMmer3.0 [17] software, and substitutions found in at least 10 genomes were chosen for further investigation. After filtering, 8 nonprotein mutations with frequencies greater than 10 were found out of 197 nonprotein mutations. A total of 3641 protein mutation features and 8 nonprotein mutation features were obtained for subsequent analysis. The first half of the name format for the protein mutation feature was the protein name, the second half was the amino acid substitution, and the number was the amino acid substitution position. Similarly, for nonprotein mutations (SNP features), the numbers represented the positions of nucleotides on the whole genome. Several studies have shown that age is an important predictor of clinical status in patients with COVID-19. Therefore, we used age after discretization as one of the essential features in our study [18,19]. In the feature matrix, a virus genome is denoted as “1” if it contains a certain feature and “0” otherwise, and this feature matrix can be found in Table S1.

### 2.2. Boruta Feature Filtering

A large number of redundant features exist among all mutation features, and they are not very helpful in distinguishing patients’ clinical status and can become noisy features for subsequent modeling. Boruta can filter the set of all features that have a correlation with the dependent variable, and the experimental results are very stable and scalable [20,21]. Here, we used Boruta for the initial filtering of mutation features, which is implemented as described below.

Boruta uses a random forest (RF) approach to extract features and disrupt the order of features to calculate the importance of features. It iteratively runs an RF on an extended version of the data for a given a dataset. The extended version of the data is a copy of the original data with horizontally connected shuffled features in each iteration. This method keeps features that are (1) more important than the best random sorting feature in each iteration and (2) superior to random factors in terms of performance (using binomial distribution). Boruta adopts the Z-Score as a measure of relevance because it considers changes in the average loss of accuracy among trees in the forest. The importance of Boruta’s approach is that it can help us gain a better understanding of the features that influence the dependent variable and, therefore, provide better and more efficient feature selection.

The Boruta program was retrieved from https://github.com/scikit-learn-contrib/boruta.py accessed on 14 September 2020 and was run with default parameters in our study.

### 2.3. Feature Ranking Algorithms

Among the features filtered by Boruta, each feature has a different importance for classification. In this study, we ranked the Boruta-filtered features using four feature ranking algorithms. Their brief descriptions are as follows.

#### 2.3.1. Max-Relevance and Min-Redundancy

Identifying features that are significant for prediction is important, but obtaining a collection of features that are not redundant is more crucial to boost robustness. When two features are strongly dependent on each other, removing one of them has little effect on the representative class’s discriminative power. As a result, the minimal redundancy requirement can be introduced to choose mutually exclusive features. The max-relevance and min-redundancy (mRMR) criteria combine the two restrictions mentioned above [22,23,24]. Initially, mRMR uses mutual information to calculate the correlation between independent variables and features, as well as between features. Ensuring maximum correlation between features and independent variables, as well as minimum redundancy between features, during the mRMR calculation process is important. 

The mRMR program from http://home.penglab.com/proj/mRMR/ accessed on 2 May 2018 was performed with default parameters in the present research.

#### 2.3.2. Monte Carlo Feature Selection

The Monte Carlo feature selection (MCFS) approach repeatedly selects many features at random before building a series of decision tree (DT) classifiers [25,26]. Intuitively, if a feature is chosen numerous times to form a classification tree, then it is significant because the classification model will choose the most distinguishing feature to become a node. A feature is given a score called relative importance depending on how well it behaves in these classifiers, which is calculated using the formula:

(1) $$RI_{g}=\sum_{\tau =1}^{st}{\left(wAcc\right)}^{u}\sum_{n_{g}\left(\tau \right)}IG\left(n_{g}\left(\tau \right)\right){\left(\frac{no.in\;n_{g}\left(\tau \right)}{no.in\;\tau }\right)}^{v},$$

where wAcc represents weighted accuracy; $IG\left(n_{g}\left(\tau \right)\right)$ represents information gain (*IG*) of $n_{g}\left(\tau \right)$; ($no.in\;n_{g}\left(\tau \right))\;$represents the sample numbers in $n_{g}\left(\tau \right)$; and $\left(no.in\;\tau \right)$ represents the sample numbers in the tree root. u and v represent two settled positive integers.

In this study, we adopted the MCFS program retrieved from http://www.ipipan.eu/staff/m.draminski/mcfs.html accessed on 4 June 2019. Such program was performed using its default parameters.

#### 2.3.3. Light Gradient Boosting Machine

The light gradient boosting machine (LightGBM) is a framework for implementing the gradient boosting decision tree algorithm which supports efficient parallel training and has the benefits of faster training speed, lower memory consumption, better accuracy, and distributed support for fast data processing [27]. The number of times a feature is used in modeling is used to determine its importance in LightGBM, and features that are used more often are considered more important. In this case, we used LightGBM in Python, which was downloaded at https://lightgbm.readthedocs.io/en/latest/ accessed on 7 May 2019 with the default parameters.

#### 2.3.4. Least Absolute Shrinkage and Selection Operator

Robert Tibshirani first presented least absolute shrinkage and selection operator (LASSO) in 1996 based on Leo Breiman’s nonnegative garrote [28,29]. It is a regularization-based regression analysis approach with a feature selection function with the goal of enhancing statistical model detection accuracy and interpretability.

In a given training sample set where $X={\left[x_{1},x_{2},\ldots \;,x_{N}\right]}^{T}\in R^{N*d}$, $x_{i}$ denotes the feature vector of the i−th sample, N denotes the number of training samples, and d denotes the feature dimension. $Y={\left[y_{1},y_{2},\ldots \;,y_{N}\right]}^{T}\in R^{N}$ denotes the classification labels corresponding to these samples. The objective function for the optimization of the LASSO feature selection method is:

(2) $$\underset{W}{\min}\frac{1}{2}{||Y-X^{T}w||}_{2}^{2}+\lambda {||w||}_{1}$$

where w denotes the regression coefficient of the eigenvector. The regularization term ${||w||}_{1}$ using the$\;L_{1}$ paradigm will produce a sparse solution in the feature space. The coefficients corresponding to irrelevant and redundant features will be set to 0, while the features corresponding to nonzero coefficients will be retained for subsequent classification. The absolute value of the regression coefficient represents the importance of the features, and the impact on the classification results is greater when the absolute value of the regression coefficient is larger. In this study, we adopted the LASSO package integrated in Scikit-learn and default parameters were used.

Through each of above algorithms, one feature list was obtained. For convenience, the lists obtained by the LASSO, LightGBM, MCFS and mRMR methods were called the LASSO, LightGBM, MCFS and mRMR feature lists, respectively.

### 2.4. Incremental Feature Selection

Four feature lists were obtained by using different feature ranking algorithms. They measured the importance of each feature in different aspects. However, the number of features in each feature list, which can be used for constructing efficient classifiers, was unknown. Here, incremental feature selection (IFS) was employed to extract the optimal subset of features for building the optimal classifiers [30,31,32]. The specific implementation steps are as follows: (1) Setting a variable k (k=1, 2, ⋯,n), where n is the number of features filtered by Boruta. (2) The top k features are taken from one feature list. (3) Samples containing k features are fed into one given classification algorithm for training a classifier, and its classification performance is assessed by ten-fold cross-validation [33]. (4) After testing all possible *k*, the classifier with the best classification performance and its corresponding features are selected as the optimal classifier and the optimal features.

### 2.5. Synthetic Minority Oversampling Technique

Synthetic minority oversampling technique (SMOTE) is an over-sampling method which can produce new samples for minor classes so as to solve the imbalanced class problem [34,35]. The main calculation procedure of the SMOTE algorithm is as follows: (1) Randomly select one sample, e.g., *x*, from the minor class. (2) Its Euclidean distances to other samples in the minor class are calculated and *k* nearest neighbors are selected. (3) One neighbor, denoted by *y*, is randomly selected from these neighbors and a new sample is calculated according to the following formula:

(3) $$x_{new}=x+rand\left(0,1\right)\times \left(y-x\right)$$

where $rand\left(0,1\right)$ stands for a random number between 0 and 1. The new sample is put into the minor class. (4) The above procedures are executed several times until samples in the minor class are same as those in the major class. In this study, the SMOTE method was employed when classifiers were constructed in the IFS method. New samples produced by SMOTE were not used in the feature analysis procedure. 

The present study adopted the SMOTE obtained from https://github.com/scikit-learn-contrib/imbalanced-learn accessed on 24 March 2020. Default parameters were used.

### 2.6. Classification Algorithm

To execute the IFS method, one classification algorithm is necessary. In this study, we tried four algorithms: DT [36], k-nearest neighbor (KNN) [37], RF [38], and support vector machine (SVM) [39]. They have wide applications in dealing with biological and medical problems [40,41,42,43,44,45,46,47,48,49].

#### 2.6.1. Decision Tree

A tree structure is referred to as a DT [36]. Each branch represents the output of this feature attribute on a value domain, and each leaf node carries a category. Starting at the root node, the associated feature attribute in the object to be classified is tested, the output branch is selected based on its value until it reaches the leaf node, and the category stored in the leaf node is taken as the decision result. This supervised learning method is based on the if-then-else rule, and the DT’s rules are learned through training rather than by hand. In this study, we use optimal DTs to extract the classification rules. We conduct DT using the Scikit-learn package and the CART method with Gini coefficients as the information gain [50]. For convenience, default parameters were used to execute the above package.

#### 2.6.2. k-Nearest Neighbor

KNN can be used for classification and regression predictive problems [37]. The closest neighbor technique uses a vector space model for categorization. The key assumption is that examples belonging to the same category have a high degree of similarity and that the categorization of cases belonging to unknown categories may be determined by comparing them to cases belonging to known categories. We achieve this precondition by specifying the default settings for the KNN model in the Scikit-learn package [50]. Likewise, default parameters were used. 

#### 2.6.3. Random Forest

RF is an ensemble learning model with strong predictive capacity that has great noise tolerance and unpredictability [38]. RF is a combinatorial classifier that relies on DTs as its foundation. This way alleviates some of the restrictions of a single classifier and allows for improved accuracy. It also alleviates some of the limitations of a single classifier and allows for improved prediction accuracy. At the same time, the unpredictability of the RF allows it to absorb the effects of outliers and noise to a higher extent, which reduces the overfitting problem of the DT method and improves generalization ability. In this case, we used RF from the Python Scikit-learn package [50]. This package was used with its default parameters. 

#### 2.6.4. Support Vector Machine

SVM is a quick and trustworthy classification system that works well with little amounts of data [39]. The principle of the SVM classification model is to use a kernel function for mapping sample points to a multidimensional feature space. Then, it constructs an optimal classification hyperplane that maximizes the distance between the hyperplane and a set of samples from different classes to maximize generalization ability. The SVM model from the Scikit-learn package is used in this research [50]. It was executed with its default parameters. 

### 2.7. Performance Evaluation

For a binary classification, the predicted results can be counted as a confusion matrix containing four entries: true positive (*TP*), false negative (*FN*), false positive (*FP*), and true negative (*TN*). Based on these entries, several measurements can be computed. In this study, we used the following measurements: sensitivity (*SN*, also called recall), specificity (*SP*), accuracy (*ACC*), *precision*, *F*1-*measure*, Matthews correlation coefficient (*MCC*), and G-Mean. They can be computed by:

(4) $$SN=\frac{TP}{TP+FN}$$

(5) $$SP=\frac{TN}{TN+FP}$$

(6) $$ACC=\frac{TP+TN}{TP+FN+TN+FP}$$

(7) $$MCC=\frac{TP\cdot TN-FP\cdot FN}{\sqrt{\left(TN+FN\right)\left(TN+FP\right)\left(TP+FN\right)\left(TP+FP\right)}}$$

(8) $$Precision=\;\frac{TP}{TP+FP}$$

(9) $$F1-measure=\frac{2\times \left(Recall\times Precision\right)}{\;\left(Recall+Precision\right)}$$

(10) $$G-mean=\sqrt{SN\cdot SP}$$

Among above measurements, the *F*1-*measure* was selected as the key measurement to help us select a classifier with the best performance. 

## 3. Results

**Figure 1:**
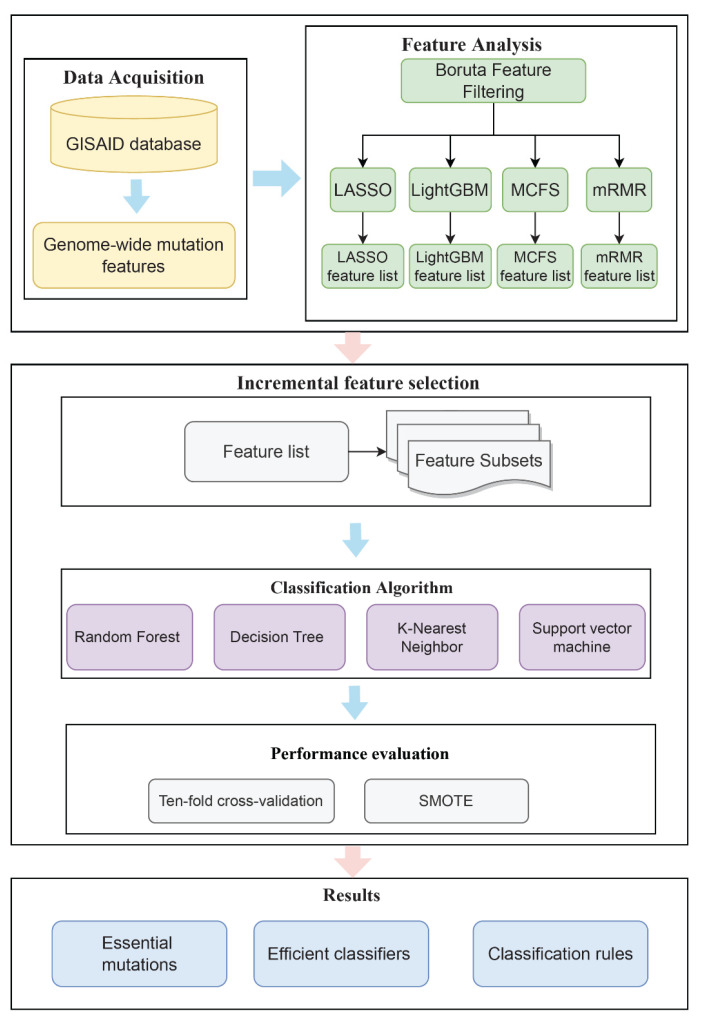
Flowchart of the whole analytical procedure of this research. Genome-wide mutation features of patients were obtained from the GISAID database and the Coronavirus Antiviral Research Database. Each patient was classified as “mild” or “severe” according to clinical status. Four lists of features were obtained after Boruta as well as four feature ranking algorithms. Subsequently, the optimal classifiers and the corresponding optimal features were obtained using the IFS method. The classification rules were mined by the optimal DT classifiers to obtain the classification basis for distinguishing the clinical status of different patients.

In this study, we extracted key mutational features to predict patients’ clinical status and identified the rules used to distinguish different clinical states by a powerful computational analysis. Figure 1 depicts the whole computing process. The outcomes of each stage would be detailed in the following sections.

### 3.1. Results of Boruta and Feature Ranking Algorithms

The Boruta method was first used to analyze the importance of all mutant features. Irrelevant features were excluded. As a result, 57 mutant features were selected by the Boruta method, which can be found in Table S2.

The obtained 57 features were further investigated by four feature ranking algorithms, respectively. Each algorithm yielded a feature list, which can also be found in Table S2.

### 3.2. Results of IFS Method on Four Feature Lists

Four feature lists generated by different feature ranking algorithms were fed into the IFS method one by one, which incorporated four classification algorithms (DT, KNN, RF and SVM). For each list, the IFS method constructed all possible feature subsets, each of which contained some top features. A classifier based on one given classification algorithm was built on each feature subset and evaluated by ten-fold cross-validation. Detailed evaluation results are listed in Table S3.

#### 3.2.1. Results of IFS Method on LASSO Feature List

**Figure 2:**
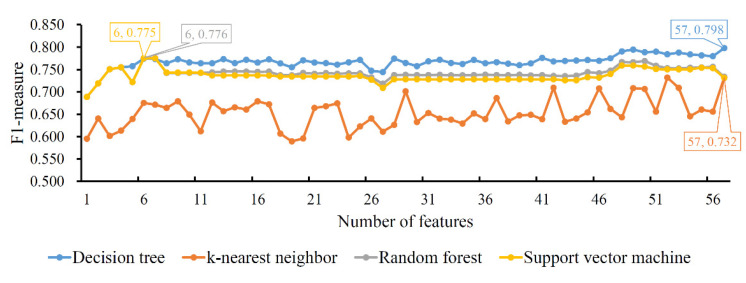
IFS curves of four classification algorithms based on the LASSO feature list. The four classification algorithms yield the highest *F*1-*measure* values of 0.798, 0.732, 0.776 and 0.775 when the top 57, 57, 6 and 6 features in the list are used.

For the LASSO feature list, the performances (*F*1-*measure*) of four classification algorithms for all possible feature subsets constructed from the list are illustrated in Figure 2. It can be observed that DT yielded the highest *F*1-*measure* of 0.798, which was obtained by using the top 57 features in the list. Accordingly, the optimal DT classifier was set up with these features. As for the other three classification algorithms, their highest *F*1-*measure* values were 0.732, 0.776 and 0.775, respectively. They were accessed by using the top 57, 6 and 6 features. Likewise, optimal KNN, RF and SVM classifiers could be built with corresponding features. Evidently, the optimal DT classifier provided a higher *F*1-*measure* than the other three optimal classifiers, indicating that it outperformed other optimal classifiers. Table 1 details the performance of the four optimal classifiers, from which we can see that the optimal DT classifier provided the highest performance on almost all measurements except *SN*. This further confirmed the superiority of the optimal DT classifier.

#### 3.2.2. Results of IFS Method on LightGBM Feature List

**Figure 3:**
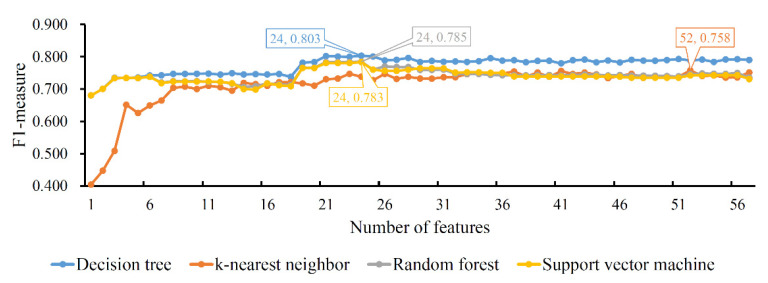
IFS curves of four classification algorithms based on the LightGBM feature list. The four classification algorithms yield the highest *F*1-*measure* values of 0.803, 0.758, 0.785 and 0.783 when the top 24, 52, 24 and 24 features in the list are used.

The same procedures were performed on the LightGBM feature list. To clearly display the performance of four classification algorithms on all feature subsets, one IFS curve was plotted for each classification algorithm, as shown in Figure 3. DT/KNN/RF/SVM yielded the highest *F*1-*measure* with 0.803/0.758/0.785/0.783. This performance was achieved by using the top 24/52/24/24 features in the list. Accordingly, an optimal DT/KNN/RF/SVM classifier could be built. Similar to the results of the LASSO feature list, the optimal DT classifier also provided the best *F*1-*measure*. By observing the measurements of the four optimal classifiers, as listed in Table 2, we could further confirm that the optimal DT classifier was better than the other three optimal classifiers.

#### 3.2.3. Results of IFS Method on MCFS Feature List

**Figure 4:**
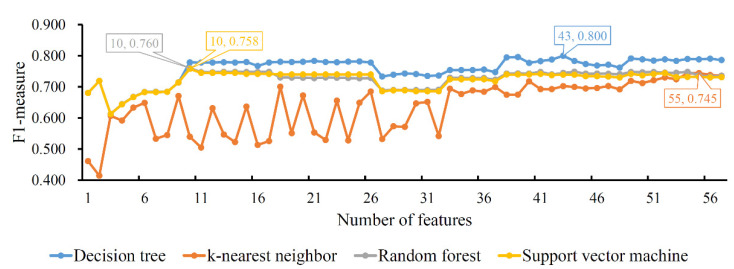
IFS curves of four classification algorithms based on the MCFS feature list. The four classification algorithms yield highest *F*1-*measure* values of 0.800, 0.745, 0.760 and 0.758 when the top 43, 55, 10 and 10 features in the list are used.

As for the MCFS feature list, the IFS method was also performed. An IFS curve was also plotted to show the performance of each classification algorithm on different feature subsets, as illustrated in Figure 4. The highest *F*1-*measure* values for four classification algorithms were 0.800, 0.745, 0.760 and 0.758, respectively, which were obtained by using the top 43, 55, 10 and 10 features in the list, respectively. Thus, we could set up the optimal DT, KNN, RF and SVM classifiers with the corresponding top features. Interestingly, the optimal DT classifier also generated the best *F*1-*measure*, conforming to the results of the LASSO and LighGBM feature lists. The performance details, including the seven measurements listed in Section 2.7, of the four optimal classifiers are provided in Table 3. It can be observed that the optimal DT classifier provided the best performance of all seven measurements, suggesting the high performance of the optimal DT classifier.

#### 3.2.4. Results of IFS Method on mRMR Feature List

**Figure 5:**
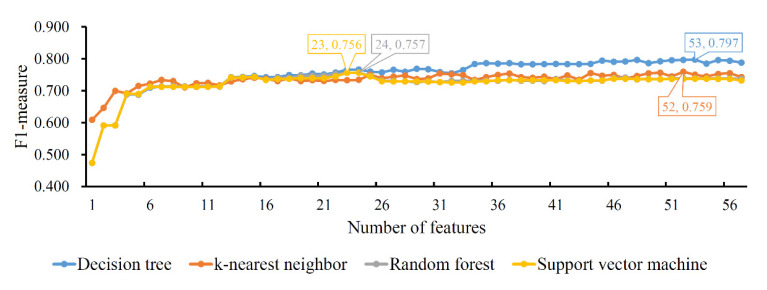
IFS curves of four classification algorithms based on the mRMR feature list. The four classification algorithms yield the highest *F*1-*measure* values of 0.797, 0.759, 0.757 and 0.756 when the top 53, 52, 24 and 23 features in the list are used.

Finally, the IFS method was applied on the mRMR feature list. The *F*1-*measure* values yielded by each classification algorithm of the different feature subsets are illustrated in Figure 5, from which we can see that the highest *F*1-*measure* values for the four classification algorithms were 0.797, 0.759, 0.757 and 0.756, respectively. The highest performance was obtained by using the top 53, 52, 24 and 23 features in the list, respectively, with which optimal DT, KNN, RF and SVM classifiers could be set up. Likewise, the optimal DT classifier also provided the best *F*1-*measure*, suggesting it was better than the other three optimal classifiers. Details of the performance of the four optimal classifiers are provided in Table 4. The optimal DT classifier generated the highest performance of the four measurements, indicating its superiority.

**Figure 6:**
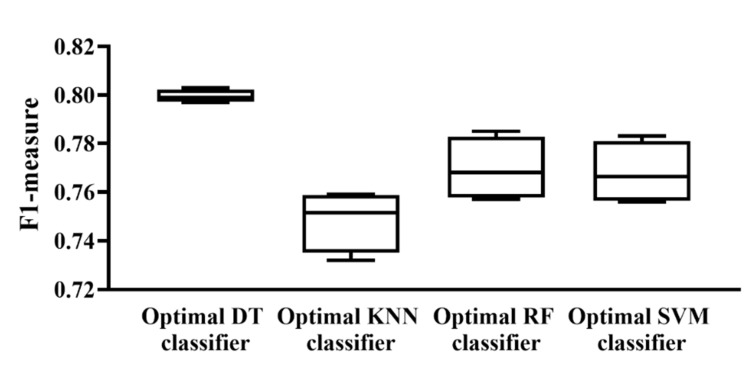
Box plot to show the performance of the optimal classifier based on different classification algorithms and feature lists. Each optimal classifier provided a similar performance in the different feature lists and the optimal DT classifier provided the highest performance.

According to the above descriptions, several optimal classifiers were set up. For each classification algorithm, four optimal classifiers were built based on four different feature lists. Their *F*1-*measure* values are illustrated in a box plot, as shown in Figure 6. It can be observed that the performance of each optimal classifier of different feature lists was quite similar and the optimal DT classifier provided the best performance. Accordingly, for each feature list, we selected the features used in the optimal DT classifiers as the optimal features.

### 3.3. Results of Intersection of Optimal Features on Different Feature Lists

**Figure 7:**
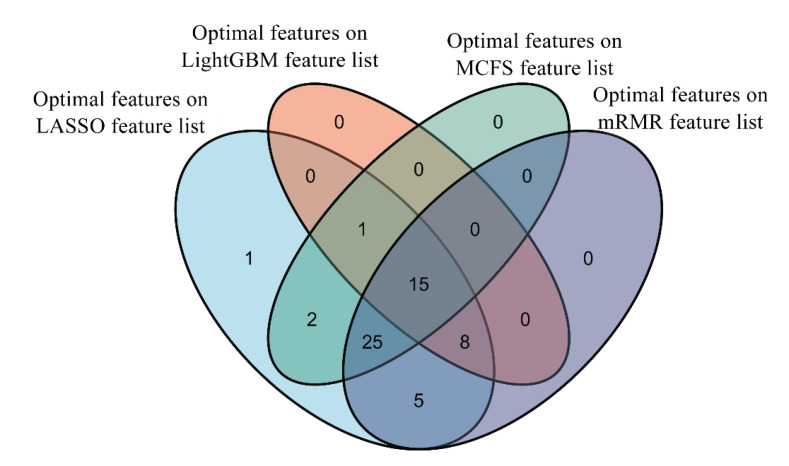
Venn diagram of the optimal feature subsets on four feature lists. Fifteen features occur in all four feature subsets, indicating their importance to differentiate the clinical status of patients.

By applying the IFS method to four feature lists, four sets of optimal features were accessed; these were the features used to construct the optimal DT classifier. The intersection of these four optimal feature subsets was investigated. Venn diagrams for these feature subsets can be found in Figure 7. It can be observed that 15 features occurred in all four feature subsets; 34 features existed in three feature subsets; 7 features belonged to exactly two feature subsets; and only 1 feature was in one feature subset. Features that appeared in four, three, two and one feature subsets are listed in Table S4. The presence of mutations in all four feature subsets indicated their importance to differentiate the clinical status of patients. A detailed explanation of these important mutations can be found in Section 4.1.

### 3.4. Classification Rules

It is known that DT is a white-box algorithm, which are always generally weaker than most black-box classification algorithms, including KNN, RF and SVM used in this study. However, as mentioned above, the optimal DT classifiers were better than the optimal KNN/RF/SVM classifier of the investigated dataset. Further investigation into such DT classifiers is helpful to reveal the relationship between mutation and the clinical status of the patients with COVID-19.

Based on the features used in each optimal DT classifier, all samples were represented by them. DT was applied on this dataset, producing a large tree from which several classification rules could be constructed. Accordingly, four sets of classification rules were obtained which included 125, 97, 89 and 126 rules. Table S5 contains a detailed description of the rules. Each rule was made up of several conditions and one result. If a sample satisfied the condition, it would be classified into the corresponding group (“mild” or “severe”). Each condition involved one mutation feature and one threshold, most of which were 0.5. This threshold indicated that the presence or absence of a set of mutations could distinguish the clinical status of patients, which suggests the association of these features and indicates that these mutations may serve as potential targets for future therapy. We further investigated these similarity rules. A detailed discussion of these rules can be found in Section 4.2.

## 4. Discussion

**Figure 8:**
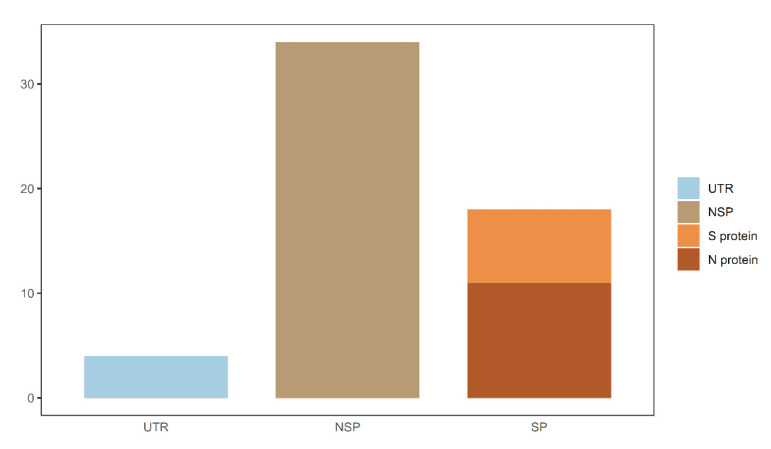
Summary of COVID-19 severity-related mutations. UTR—untranslated region; NSP—nonstructural protein; SP—structural protein; S protein—spike protein; N protein—envelope protein.

**Figure 9:**
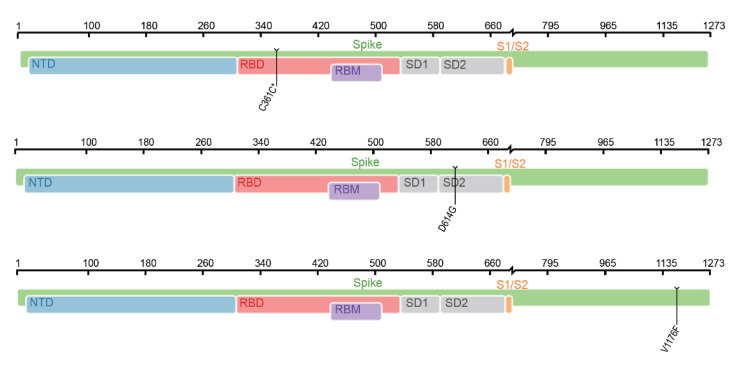
Location of some spike protein mutations in the genome. NTD—N-terminal domain; RBD—receptor-binding domain; RBM—receptor-binding motif; SD1—subdomain 1; SD2—subdomain 2; S1/S2—S1/S2 cleavage region.

In this study, data related to COVID-19 were deeply investigated by several computational algorithms. Four sets of optimal mutation features and decision rules that were strongly correlated with the severity of COVID-19 were identified. By combining features in four subsets, 57 mutation features were obtained which were deemed to be COVID-19 severity-related. Except the feature of “age”, we classified the remaining 56 mutations into distinct categories according to the genomic locations and protein functions. As shown in Figure 8, mutations in both untranslated regions and gene regions were associated with COVID-19 severity. The locations of some important spike protein mutations in the genome are shown in Figure 9. The analysis of these mutational signatures and decision rules may inform clinical evaluation and vaccine development.

### 4.1. Features Associated with COVID-19 Severity

We observed that 15 features (i.e., V1176F, age, ORF3a:Q57H, RdRP:P323L, N:I292T, nsp14:A320V, ORF6:I33T, SNP:C241T, nsp4:F308Y, nsp7:L71F, C361C*, D614G, nsp6:L37F, N:S202N, and nsp14:I164ILV) were simultaneously present in the results of the four groups involving age, S protein mutations, SNP mutations in the UTR, and mutations in other proteins. Most of these mutations have been widely reported to be associated with the pathogenicity of SARS-CoV-2, while some mutations have been less studied (e.g., nsp14:A320V, ORF6:I33T, C361C*, and N:S202N).

Among the 15 most significant mutational features associated with COVID-19 severity, V1176F is a classical mutational signature of the S protein and was originally identified in the Gamma variant. Studies suggest that V1176F may enhance S protein flexibility and is associated with higher patient mortality [51,52]. Another important S protein mutation in our results was D614G, and previous studies have shown that the mutation of D614G leads to the binding affinity of the virus to ACE2, thus increasing the efficiency of cell entry and further enhancing the infectivity of the virus [53]. Although previous studies of hospitalization outcomes have shown no significant association between D614G and disease severity, our results show that it is important for post-COVID-19 severity prediction [54]. Another S protein mutation, C361C*, is also associated with COVID-19 severity, which has not been investigated in depth in previous studies.

Numerous mutations in other encoded proteins have also been shown to correlate with disease severity. For example, nsp4:F308Y and nsp6:L37F have been reported to be associated with mild disease, while ORF3a:Q57H, RdRP:P323L, N:I292T, ORF6:I33T, and nsp7:L71F are associated with severe disease [55]. N:S202N is a hot spot mutation in a linker region which may be associated with enhanced RNA binding and altered serine phosphorylation, and no evidence has shown that N:S202N is associated with disease severity in COVID-19 patients. Our results also show that nsp14:A320V and nsp14:I164ILV are associated with the disease severity, which has not been reported in previous studies. Nsp14 is known to function in viral replication and transcription. Mutations in nsp14 may cause the alterations of viral functions, thus affecting virological properties and disease severity.

Age is also an important affecting factor of disease progression, and the infection rate of COVID-19 and the risk of serious disease increase with rising age [56,57]. Another important mutation with a small proportion is SNP:C241T in the UTR region. It is often accompanied with RdRP:P323L or D614G variants, and no studies have directly shown that SNP:C241T is associated with the severity of COVID-19 [58].

By combining existing studies, we found that 15 features are important for identifying the severity of COVID-19. This deduction not only demonstrates the reliability of our study, but also suggests that some under-appreciated mutations (e.g., C361C*, N:S202N, nsp14:A320V, nsp14:I164ILV, and SNP:C241T) may be associated with COVID-19 severity.

### 4.2. Decision Rules Related to the Severity of COVID-19 Infections

The four sets of decision rules (Table S5) were also similar, and we further analyzed the rules with higher passed counts to classify the severity of COVID-19 for most samples according to different mutations.

Our decision rules often involved a combination of multiple criteria, but we found that a combination of two genes (i.e., RdRP:P323L and V1176F) could partly determine cases of severe COVID-19 disease, and that the decision rules related to the results of the four groups of algorithms. This finding is consistent with that of existing studies; specifically, RdRP:P323L and V1176F are associated with poorer clinical presentation [52,55].

We also found that RdRP:P323L is often accompanied by ORF3a:Q57H, D614G, SNP:C241T, nsp2:T85I, and other mutations in severe COVID-19 patients. D614G is a hotspot mutation that appeared as early as the Alpha variant, and although the D614G mutation does not directly correlate with disease severity, other COVID-19 variants containing this mutation may accelerate infection due to the ability to facilitate viral entry into cells and further aggravate the disease [54,59]. ORF3a:Q57H and nsp2:T85I first appeared in the Beta variants and our results suggest that they may be associated with severe outcomes of SARS-CoV-2 infection [60]. Studies have shown that SNP:C241T mutation is associated with lower viral replication efficiency [61] and it can be observed in our results that mild patients with SNP:C241T mutation tend to be combined with less other severe mutation features (e.g., nsp2:T85I, ORF3a:Q57H, and V1176F), which suggests that this gene mutation may be indirectly associated with mild COVID-19.

In our results, some COVID-19 mutations associated with mild disease were also found, such as nsp14:I164ILV, ORF8:L84S, nsp4:F308Y, nsp6:L37F, ORF3a:G196V, N:P13L, nsp4:M324I, and N:G204R. These mutations tend to be present in low-risk patients with different combinations, and some have been reported to be associated with mild disease or disease transmissibility [55,60].

### 4.3. Comparsion of the Previous Study

In Nagy et al.’s study [15], a computation analysis was also conducted on similar datasets. However, there were several differences between our study and theirs. The main differences were as follows.

(1) The mutation features analyzed in our study were generated by a different platform. As different platforms have different advantages and disadvantages, analyzing mutation features obtained from different platforms can uncover novel mutations that are highly correlated with the clinical status of patients with COVID-19;
(2) More machine learning algorithms were used in this study than in Nagy et al.’s study. We used five feature analysis methods (Boruta, LASSO, LightGBM, MCFS and mRMR) and four classification algorithms (DT, KNN, RF and SVM). With these algorithms, each mutation feature was fully evaluated. The final mutation features were selected by multiple feature analysis methods, increasing the reliability of the results;
(3) In this study, we not only discovered mutation features related to the clinical status of the patients with COVID-19 but also established rules to indicate more complicated mutation patterns of the clinical status of the patients with COVID-19. These patterns always included multiple mutation features, suggesting the relationships between a combination of mutation features and the clinical status of patients with COVID-19. Such form can be deemed an extension of single mutation biomarkers, and was not involved in Nagy et al.’s study;
(4) Biological analysis was performed in our study, increasing the reliability of the results. In Nagy et al.’s study, the important mutation features were only listed and were not analyzed.

These differences indicate that the new findings reported in this study can provide novel insights to investigate the relationships between mutation and the clinical status of patients with COVID-19.

## 5. Conclusions

In this research, we utilized sophisticated and widely used computational approaches in relation to genome-wide mutation data to explore important mutations used to distinguish clinical status in COVID-19 patients. In short, our results demonstrated a set of mutations associated with SARS-CoV-2 severity, which could help rapidly identify SARS-CoV-2 infections carrying mutations associated with severe outcomes and guide the development of SARS-CoV-2 vaccines. We also suggested some effective classifiers for predicting COVID-19 patients’ clinical status; these were trained with a vast quantity of data and performed well in classification. The quantitative decision rules in the optimal DT classifier provided direct clues to distinguish between different patient clinical states.

## Figures and Tables

**Table 1:** Performance of the optimal classifiers based on different classification algorithms and LASSO feature list ^1^.

Term	Decision Tree	k-Nearest Neighbor	Random Forest	Support Vector Machine
Number of features	57	57	6	6
*SN*	0.788	**0.824**	0.821	0.820
*SP*	**0.832**	0.616	0.734	0.734
*ACC*	**0.811**	0.714	0.775	0.775
*MCC*	**0.621**	0.447	0.555	0.554
*Precision*	**0.808**	0.658	0.735	0.735
*F*1-*measure*	**0.798**	0.732	0.776	0.775
G-mean	**0.809**	0.712	0.776	0.776

^1^ Numbers in bold are highest in the corresponding rows.

**Table 2:** Performance of the optimal classifiers based on different classification algorithms and LightGBM feature list ^1^.

Term	Decision Tree	k-Nearest Neighbor	Random Forest	Support Vector Machine
Number of features	24	52	24	24
*SN*	0.844	**0.878**	0.813	0.816
*SP*	**0.769**	0.605	0.768	0.760
*ACC*	**0.804**	0.734	0.789	0.787
*MCC*	**0.612**	0.498	0.580	0.575
*Precision*	**0.766**	0.666	0.759	0.754
*F*1-*measure*	**0.803**	0.758	0.785	0.783
G-mean	**0.805**	0.729	0.790	0.788

^1^ Numbers in bold are highest in corresponding rows.

**Table 3:** Performance of the optimal classifiers based on different classification algorithms and MCFS feature list ^1^.

Term	Decision Tree	k-Nearest Neighbor	Random Forest	Support Vector Machine
Number of features	43	55	10	10
*SN*	**0.848**	0.832	0.816	0.811
*SP*	**0.755**	0.637	0.704	0.704
*ACC*	**0.799**	0.730	0.757	0.755
*MCC*	**0.603**	0.476	0.521	0.516
*Precision*	**0.757**	0.673	0.712	0.711
*F*1-*measure*	**0.800**	0.745	0.760	0.758
G-mean	**0.800**	0.728	0.758	0.756

^1^ Numbers in bold are highest in corresponding rows.

**Table 4:** Performance of the optimal classifiers based on different classification algorithms and the mRMR feature list ^1^.

Term	Decision Tree	k-Nearest Neighbor	Random Forest	Support Vector Machine
Number of features	53	52	24	23
*SN*	0.777	**0.885**	0.666	0.665
*SP*	0.846	0.598	**0.915**	0.916
*ACC*	**0.813**	0.734	0.797	0.797
*MCC*	**0.625**	0.501	0.604	0.604
*Precision*	0.819	0.665	**0.875**	0.877
*F*1-*measure*	**0.797**	0.759	0.757	0.756
G-mean	**0.810**	0.728	0.781	0.780

^1^ Numbers in bold are highest in the corresponding rows.

## Data Availability

The data presented in this study are openly available at https://www.gisaid.org/ (accessed on 6 December 2021).

## Supplementary Materials

The following supporting information can be downloaded at: https://www.mdpi.com/article/10.3390/life12060806/s1, Table S1: SARS-CoV-2 genomic feature matrix. Rows represent samples, and columns represent mutational features. In the feature matrix, a virus genome is denoted as “1” if it contains a certain feature and “0” otherwise, Table S2: Feature list filtered by Boruta and ordered by four feature ranking algorithms, Table S3: Performance of IFS with different classifiers, Table S4: Intersection results of the optimal feature subsets in the four ranking algorithms. The column names represent features appearing in n (n = 4,3,2,1) feature sorting algorithms, Table S5: Classification rules generated by the optimal DT model based on four ranking algorithms.
